# Protective Effect of Low Molecular Weight Peptides from *Solenocera crassicornis* Head against Cyclophosphamide-Induced Nephrotoxicity in Mice *via* the Keap1/Nrf2 Pathway

**DOI:** 10.3390/antiox9080745

**Published:** 2020-08-13

**Authors:** Shuoqi Jiang, Zhuangwei Zhang, FangFang Huang, Zuisu Yang, Fangmiao Yu, Yunping Tang, Guofang Ding

**Affiliations:** Zhejiang Provincial Engineering Technology Research Center of Marine Biomedical Products, School of Food and Pharmacy, Zhejiang Ocean University, Zhoushan 316022, China; S18070700050@zjou.edu.cn (S.J.); zw.zhang1997@zjou.edu.cn (Z.Z.); gracegang@126.com (F.H.); yzs@zjou.edu.cn (Z.Y.); fmyu@zjou.edu.cn (F.Y.)

**Keywords:** apoptosis, cyclophosphamide, inflammation, oxidative stress, peptides, *Solenocera crassicornis*

## Abstract

The major component of the *Solenocera crassicornis* head protein hydrolysates-fraction 1 (SCHPs-F1) are low molecular weight peptides (MW < 1 kDa). In this study, we investigated the potential renoprotective effects of SCHPs-F1 in a cyclophosphamide (CTX) toxicity mouse model. In brief, 40 male mice were randomly divided into 5 groups and received either saline or 80 mg/kg body weight (BW) CTX by intraperitoneal injection for 5 days, followed by either saline or SCHPs-F1 (100, 200, and 400 mg/kg BW) by intragastric administration for 15 days. SCHPs-F1 treatment significantly reversed the CTX-induced decreases in the levels of blood urea nitrogen (BUN), creatinine (CRE), and cytochrome P450 (CYP450), as well as the renal histological lesions. Furthermore, the results indicated that SCHPs-F1 potentially alleviated CTX-induced nephrotoxicity through mitigating inflammatory responses, oxidative stress, and apoptosis status of the kidneys, as evidenced by decreased levels of malondialdehyde (MDA), interleukin (IL)-1β, IL-6, tumor necrosis factor (TNF)-α, and interferon (IFN)-γ and increased levels of total antioxidant capacity (T-AOC), catalase (CAT), superoxide dismutase (SOD) and glutathione peroxidase (GSH-Px). Moreover, overexpression of pro-apoptotic proteins pair B-cell lymphoma-2 (Bcl-2)-associated X (Bax)/Bcl-2, cysteinyl aspartate specific proteinase (caspase)-3 and caspase-9 in renal tissues were suppressed by treatment with SCHPs-F1. In addition, the protein levels of the antioxidant factor nuclear factor erythroid-2 related factor 2 (Nrf2) and the expression levels of its downstream target genes heme-oxygenase (HO-1), glutamate-cysteine ligase modifier subunit (GCLM) and NAD(P)H dehydrogenase (quinone) 1 (NQO-1) were stimulated by treatment with SCHPs-F1 in the CTX-induced renal injury model. Taken together, our data suggested that SCHPs-F1 could provide a novel potential strategy in mitigating the nephrotoxicity caused by CTX.

## 1. Introduction

Cyclophosphamide (CTX) is the most extensively used drug in clinical cancer chemotherapy, with a high therapeutic index and broad-spectrum anti-cancer effect. As a prodrug, CTX needs to be converted into active metabolites to properly function *in vivo* [1]. However, the multiple organ toxicity caused by its metabolites has seriously limited the role of CTX in the comprehensive treatment of cancer [2]. The mechanism underlying the multiple organ toxicity of CTX has been examined in numerous studies, and it has been found to correlate with oxidative stress, inflammation, and apoptosis [3,4,5]. CTX exposure perturbs the redox balance and exhausts the antioxidant defense system of the kidneys, resulting in oxidative damage to the renal tissue. In addition, inflammatory cascades were subsequently activated and involved in the up-regulation of apoptosis, leading to nephrocyte necrosis and apoptosis, disorganization of renal tissue, and renal dysfunction [6,7,8,9]. Therefore, alleviating oxidative stress, inflammation, and the apoptosis status of renal tissue may serve as a therapeutic strategy for CTX-induced nephrotoxicity.

*Solenocera crassicornis* is a marine animal with abundant resources, and its great economic value is reflected in the shrimp meat that is rich in protein and minerals [10]. The tails are destined to become shelled shrimp, whereas plenty of shrimp heads are discarded as processing leftovers. It has previously been reported that the waste of shrimp heads produced in commercial development accounts for roughly 40% of the shrimp body weight, which is a potential protein source that can be used for producing bioactive functional components [11]. Several studies have been executed to prepare protein hydrolysates and bioactive peptides of varying sizes using industrial waste from various shrimp processing leftovers [11,12,13]. To further assess commercial high-value applications of *Solenocera crassicornis* shrimp heads, we prepared low molecular weight peptides (MW less than 1 kDa, SCHPs-F1) and found they could effectively alleviate CTX-induced hepatotoxicity in mice [14]. Based on the toxicological and metabolic mechanism of action of CTX that involves the induction of multiple organ toxicity [8], we speculated that SCHPs-F1 supplementation may also have the potential to alleviate CTX-induced nephrotoxicity.

Therefore, we investigated the potential protective effect of SCHPs-F1 through the intervention of SCHPs-F1 in a mouse model of CTX-induced renal injury, evaluated renal function markers, and performed histopathology analyses. In addition, we investigated the relationship between oxidative stress, inflammation, and apoptosis in renal cells and the improvement of renal injury and renal function. The results indicate that SCHPs-F1 has the potential to alleviate CTX-induced renal injury in mice and that the possible underlying molecular mechanism involves the up-regulation of the Nrf2 antioxidant signaling pathway.

## 2. Materials and Methods

### 2.1. Chemicals and Reagents

*Solenocera crassicornis* was purchased from the Zhoushan International Aquatic Center (Zhoushan, Zhejiang, China). Pepsin, trypsin, 2,2-Di(4-tert-octylphenyl)-1-picrylhydrazyl (DPPH^•^), 1, 10-Phenanthroline, Nitrotetrazolium Blue chloride, Nicotinamide adenine dinucleotide hydrate, and Phenazine methosulfate were purchased from Sigma-Aldrich Trading Co., Ltd. (Shanghai, China). CTX was purchased from Aladdin Bio-Chem Technology Co., Ltd. (Shanghai, China). Hematoxylin and Eosin (H & E) staining kit and Modified Masson trichrome staining kit were provided by Nanjing SenBeiJia Biological Technology Co., Ltd. (Nanjing, China). Terminal deoxynucleotidyl transferase dUTP nick end labeling (TUNEL) apoptosis detection kit (Alexa Fluor 640) was purchased from Yeasen Biotech Co. Ltd. (Shanghai, China). Antibodies directed against Bcl-2 (CST3498S, 1:2000) and Bax (CST14796S, 1:2000) were purchased from Cell Signaling Technology (Danvers, MA, USA); antibodies against Nrf2 (AF7623, 1:1000), Keap1 (AF7335, 1:1000), NQO-1 (AF7614, 1:1000), HO-1 (AF1333, 1:1000), GCLM (AF6972, 1:1000), and β-actin (AF5001, 1:1000) were purchased from Beyotime Biotechnology (Shanghai, China); and caspase-3 (K003262P, 1:1000), caspase-9 (K008077P, 1:1000), goat anti-rabbit IgG/HRP antibody (SE134, 1:1000), and goat anti-mouse IgG/HRP antibody (SE131, 1:1000) were purchased from Solarbio Sci-technology Co. Ltd. (Beijing, China). Ultrapure water was obtained using a Milli-Q water purification system from Millipore (Bedford, MA, USA). Other chemical reagents were purchased from Sinopharm Chemical Reagent Co., Ltd. (Shanghai, China).

### 2.2. Preparation of SCPHs-F1

SCHPs-F1 was prepared according to the process described in our previous report [14]. In brief, defatted shrimp heads were homogenized and allowed to react in a pH-adjusted (pH = 3.0) pepsin-containing aqueous solution (2500 U/g, *m/m* = 1:10) for 4 h. Then, the pH was adjusted to 8.0 under optimal trypsin conditions (2500 U/g) in a reaction system for 4 h. The solution was heated to inactivate the enzyme, and the supernatant was collected (CF16RN high-speed microcentrifuge, Himac, Tokyo, Japan), filtered through a microfilter (0.22 μm), and ultrafiltered using a 1 kDa membrane. The supernatant (<1 kDa) obtained by ultrafiltration (GM-18 roll film separation system, Bona Biotechnology Co., Ltd., Jinan, China) was freeze-dried (Christ Alpha 1–4 LD plus Laboratory freeze dryer, Marin Christ, Osterode, Germany) to prepare SCHPs-F1 for future studies.

### 2.3. Antioxidants Activity of SCHPs-F1

#### 2.3.1. Free Radical Scavenging

The protocols of DPPH^•^, ^•^OH, and O_2_^•^^−^ radical scavenging assays were executed as described in our previous reports [15,16]. The concentration for 50% of maximal radical scavenging (EC_50_) was calculated with the “Quest Graph™EC_50_ Calculator” [17]. In the positive control group, the SCPHs-F1 samples were substituted with reduced-glutathione (GSH).

- *DPPH^•^ Scavenging Activity:* SCPHs-F1 samples at indicated concentrations (0.1, 0.25, 0.5, 1, 2, 4, and 8 mg/mL) were prepared in ultrapure water (water only for the control sample). Next, 0.2 mM DPPH (prepared by ethanol) work solution was added to the above solutions (ethanol only for the blank control). All mixtures were reacted at room temperature in the dark for 30 min, and the absorbance (A) of solutions was recorded with UV-vis spectra (SpectraMax M2, Molecular Devices Co., San Jose, CA, USA) at 517 nm. The DPPH^•^ scavenging activity of each sample was computed with the following equation:

DPPH^•^ radical scavenging activity (%) = (A _Control_ + A _Blank control_ − A _Sample_)/A _Control_ × 100 (1)

^•^*OH Scavenging Activity:* The above samples were mixed with 1, 10-Phenanthroline work solution (1.0 mL, 1.865 mM), FeSO_4_·7H_2_O (1.0 mL, 1.865 mM), and H_2_O_2_ (1.0 mL, 0.03%, *v/v*) respectively (control without samples, blank control without H_2_O_2_). After reaction at 37 °C for 1 h, the absorbance of solutions was recorded with UV-vis spectra at 536 nm. The ^•^OH scavenging activity of each sample was computed with the following equation:

^•^OH radical scavenging activity (%) = (A _Sample_ − A _Control_)/(A _Blank control_ − A _Control_) × 100 (2)

- *O_2_^•^^−^ Scavenging Activity:* The above samples were mixed with Nitrotetrazolium Blue chloride (1.0 mL, 2.52 mM), Nicotinamide adenine dinucleotide (1.0 mL, 624 mM), and Phenazine methosulphate (1.0 mL, 120 μM), respectively (control without samples). After reaction at room temperature in the dark for 5 min, the absorbance of solutions was recorded with UV-vis spectra at 560 nm. The O_2_^•^^−^ scavenging activity of each sample was computed with the following equation:

O_2_^•^^−^ radical scavenging activity (%) = (A _Control_ − A _Sample_)/A _Control_ × 100 (3)

#### 2.3.2. Reducing Power

The reducing power assay was performed according to our laboratory standard protocol [18]. The above samples were mixed with potassium hexacyanoferrate (2.5 mL, 1%, *m/v*) and reacted at 50 °C for 30 min. Then, trichloroacetic acid (1.5 mL, 10%, *v/v*) was added to mixtures, and 2.0 mL of the upper layer of mixtures was mixed with ultrapure water (2.0 mL) and FeCl_3_ (0.5 mL, 0.1%, *m/v*). Next, the absorbance of each solution was recorded with UV-vis spectra at 700 nm.

### 2.4. Renoprotective Effects on CTX-Induced Mice

#### 2.4.1. Animals and Experimental Plan

**Figure 1:**
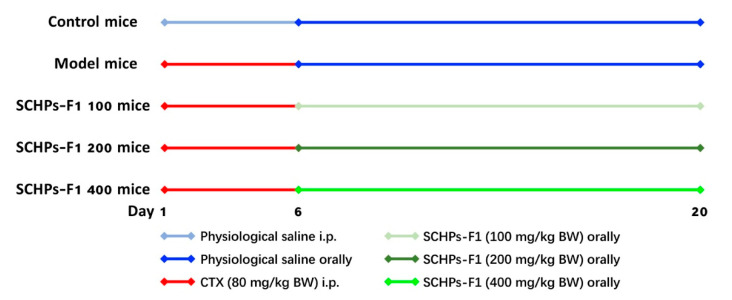
Outline of the experimental protocol regarding the treatment of the various groups.

Male ICR mice (6 weeks old; weight, 18–22 g; n = 40) were purchased from the Zhejiang Lab-animal Public Service Platform (specific pathogen-free (SPF) Grade, Certificate No. SCXK-ZHE 2014-0001, Hangzhou, China) and acclimated for 1 week in an SPF environment that was maintained at a temperature of 22 ± 2 °C and relative humidity of 50–60%. Mice were fed commercial mouse chow and sterile water in a 12:12 h day and night cycle. Experimental procedures were approved by the Animal Ethics Committee of the Committee for Research Ethics and Integrity of Zhejiang Ocean University (Zhoushan, Zhejiang, China, No. SCXK ZHE 2019-0031) and complied with the regulations on all ethical and legal requirements of experimental animals in the guidelines for the care and use of experimental animals published by the National Institutes of Health (Bethesda, MD, USA). After habituation, mice were randomly assigned to five groups (*n* = 8) and treated as indicated in Figure 1.

The body weight (BW) of all animals was recorded and the renal index (organ weight/body weight) was calculated. During the entire course of the experiment, the mortality rate was zero.

#### 2.4.2. Sample Collection

After the last SCHPs-F1 treatment, all mice were euthanized by cervical dislocation. Blood was collected through retro-orbital bleeding and used for analyzing biochemical parameters in serum (centrifugation at 500× *g* for 10 min). The kidneys were rapidly excised, rinsed in cold 0.1 M PBS (pH = 7.4), and weighed. Part of the renal tissue was pre-cooled in 0.1 M PBS (*w/v*, 1:9), homogenized (D-500 homogenizer, Dragon Lab, Beijing, China), and centrifuged to obtained supernatant for evaluating the renal function and inflammatory and oxidative parameters. In addition, for each group of mice, parts of renal tissue were combined and powdered under liquid nitrogen, and then lysed with radioimmunoprecipitation assay (RIPA) lysis buffer (Beyotime Institute of Biotechnology, Shanghai, China) on ice. The lysate’s supernatant was collected and total protein concentration was estimated using bicinchoninic acid (BCA) protein assay kit (Beijing Solarbio Science & Technology Co., Ltd., Beijing, China).

#### 2.4.3. Biochemistry Assays

Renal function parameters: Serum was prepared from the blood that was obtained through retro-orbital bleeding to measure the renal function markers blood urea nitrogen (BUN) and creatinine (CRE) (Nanjing Jiancheng Bioengineering Institute, China). The supernatant of renal homogenates was used for the detection of cytochrome P450 (CYP450) by an Enzyme-Linked Immunosorbent Assay (ELISA) kit (Nanjing SenBeiJia Biological Technology Co., Ltd., Nanjing, China).

Oxidative parameters: The activities of catalase (CAT), total antioxidant capacity (T-AOC), superoxide dismutase (SOD), malondialdehyde (MDA), and glutathione peroxidase (GSH-Px) in renal tissue homogenates were determined using diagnostic kits (Nanjing Jiancheng Bioengineering Institute, Nanjing, China).

Inflammatory parameters: The levels of pro-inflammatory cytokines interleukin (IL)-1β, IL-6, tumor necrosis factor (TNF)-α, and interferon (IFN)-γ in renal homogenates were analyzed by ELISA kits (Boster Biological Technology Co., Ltd., Wuhan, China).

All assays were read on a SpectraMax M2 multi-wavelength strip reader (Molecular Devices, San Jose, CA, USA) following the manufacturer’s guidelines.

#### 2.4.4. Histopathological Examination

A portion of the renal tissue was collected and fixed in 4% paraformaldehyde. Paraffin blocks were prepared, 4-μm paraffin sections were cut, and conventional dewaxing to water was performed (xylene dewaxing twice for 5 min; gradual rehydration with 100%, 90%, 80%, 70% gradient alcohol and distilled water for 3 min [19]). H&E staining and Masson trichrome staining were performed as described in the commercial kit instructions. Micrographs were obtained using a Biological microscope CX31 (Olympus, Tokyo, Japan).

For the TUNEL assay, paraffin sections of kidney tissue were immersed in xylene twice for 5 min and paraffin was removed completely. Then, the sections were soaked in gradient ethanol (100, 90, 80, 70%) for 3 min at room temperature. Residual alcohol was removed by PBS, and the contour of sample distribution was drawn with a crayon pen. Finally, the TUNEL staining was performed according to the manufacturer’s instructions (Yeasen Biotech Co. Ltd., Shanghai, China). Micrographs were obtained using an Axio Imager A2 fluorescence microscope (Carl Zeiss, Oberkochen, Germany).

#### 2.4.5. Western Blot Analysis

Western blot analysis was performed according to routine laboratory methods [20]. In brief, non-fat powdered milk was used to block non-specific antigens on PVDF membranes. Then, membranes were incubated with primary antibodies (Keap1, Nrf2, HO-1, GCLM, NQO-1, Bax, Bcl-2, caspase-3, caspase-9, and β-actin) at 4 °C overnight. After binding with primary antibodies, membranes were washed three times and then incubated with an HRP-labeled secondary antibody at room temperature for 1 h. For visualization, chemiluminescence (Enhanced chemiluminescence western lightning kit, Beyotime Institute of Biotechnology, Shanghai, China), imaging (FluorChem FC3 gel imaging analysis system, ProteinSimple, Silicon Valley, CA, USA) and quantification (AlphaView software, version 3.4.0, ProteinSimple) were employed.

#### 2.4.6. Statistical Analysis

Numerical results were expressed as the mean ± standard deviation. Statistically significant differences between groups were conducted using the analysis of variance (ANOVA) function of the Statistical Product and Service Solutions software, version 24.0 (SPSS Inc., Chicago, IL, USA).

## 3. Results

### 3.1. Radical Scavenging Activity of SCHPs-F1

**Figure 2:**
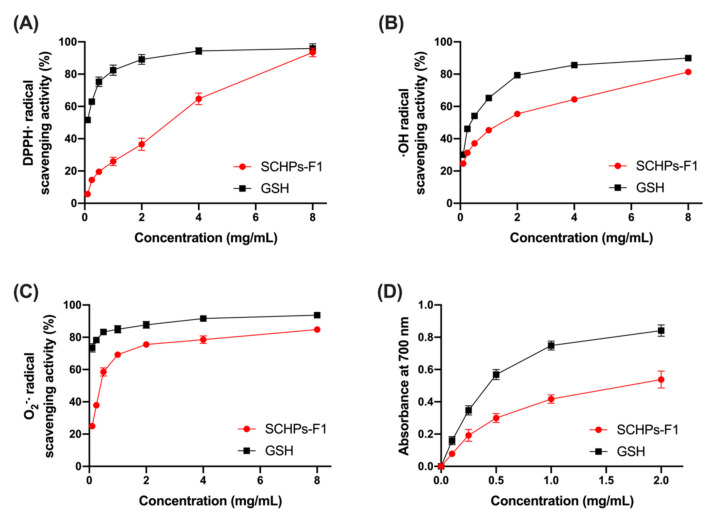
Antioxidant activity *in vitro* of SCHPs-F1 determined by DPPH^•^ (**A**), ^•^OH (**B**), and O_2_^•^^−^ (**C**) scavenging activities, and ferric ion-reducing power (**D**) of SCHPs-F1. Reduced glutathione (GSH) serves as a positive control.

To investigate the antioxidant activity of SCHPs-F1 *in vitro*, the free radical scavenging and ferric ion-reducing power assays were performed. Within the concentration range of 0.1–8 mg/mL, the co-incubation of increasing concentrations of SCHPs-F1 caused a general trend of increasing radical scavenging ratio. The EC_50_ values of SCHPs-F1 were 5.16 mg/mL, 2.17 mg/mL, and 0.37 mg/mL for scavenging DPPH^•^, ^•^OH, and O_2_^•^^−^, respectively (Figure 2A–C). The results of ferric ion-reducing antioxidant power assay are shown in Figure 2D, the increasing absorbance values of the reaction system corresponded to the reducing power of SCHPs-F1. There is a positive correlation between SCHPs-F1 concentrations and the absorbance values, wherein SCHPs-F1 reduced more Fe (III) to Fe (II), and further formed Prussian blue with ferric chloride.

### 3.2. SCHPs-F1 Treatment Modulates Renal Function in Mice Exposed to CTX

**Figure 3:**
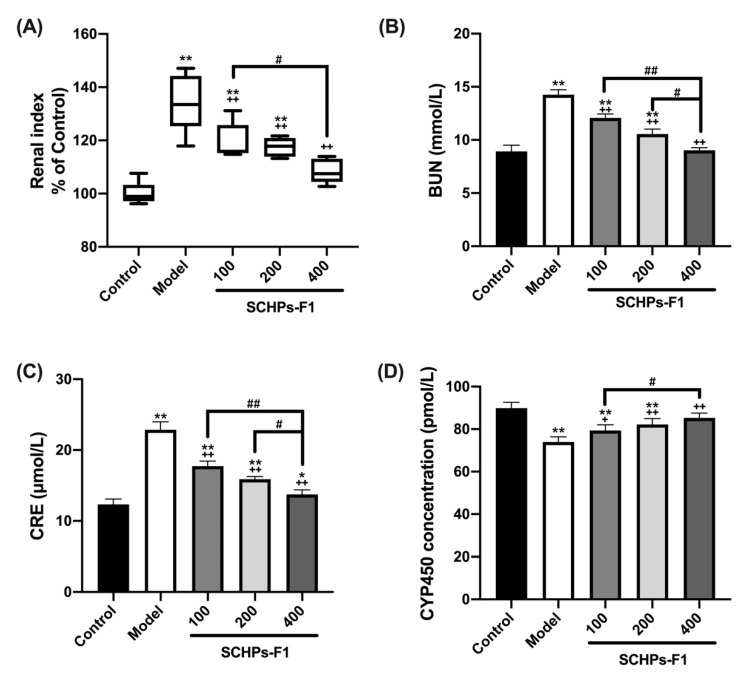
Effects of SCHPs-F1 on renal index (**A**) and renal function parameters of BUN (**B**), CRE (**C**), and CYP450 (**D**) levels in CTX-induced mice. * *p* < 0.05, ** *p* < 0.01, vs. control group; + *p* < 0.05, ++ *p* < 0.01, vs. model group; # *p* < 0.05 and ## *p* < 0.01 indicate significant differences between different SCHPs-F1 dose groups.

As shown in Figure 3A, the renal index of CTX-treated mice was dramatically increased compared with the control group (*p* < 0.01). However, treatment with SCHPs-F1 significantly restored the CTX-induced increase of the renal index, and mice post-treated with 400 mg/kg SCHPs-F1 presented similar levels to the control group (*p* > 0.05).

The effect of SCHPs-F1 treatment on renal function parameters is presented in Figure 3B–D. CTX caused renal dysfunction, which was reflected in renal function parameters, such as an increase in the most sensitive parameters as compared with the control group, including serum BUN and CRE for assessment of the function of glomerular filtration. The abnormal increase of BUN (14.27 ± 0.46 mmol/L) and CRE (22.86 ± 1.13 μmol/L) caused by CTX was fully suppressed after treatment with 400 mg/kg SCHPs-F1 (9.02 ± 0.25 mmol/L and 17.73 ± 0.67 μmol/L, respectively, *p* < 0.01).

Furthermore, the renal metabolic system CYP450 was significantly reduced in response to CTX. Mice treated with 100 mg/kg, 200 mg/kg and 400 mg/kg SCHPs-F1 had significantly increase in CYP450 concentrations (79.41 ± 2.64 pmol/L, 82.22 ± 2.75 pmol/L and 85.26 ± 2.27 pmol/L, respectively) compared to CTX-treated mice (73.99 ± 2.36 pmol/L). No significant differences were observed between mice treated with 400 mg/kg SCHPs-F1 and the control group (*p* > 0.05).

### 3.3. SCHPs-F1 Treatment Relieves CTX-Induced Renal Oxidative Stress

Next, we investigated the effect of SCHPs-F1 on the lipid peroxidation indicator MDA, the antioxidant activities of GSH-Px, SOD, and CAT, as well as T-AOC levels in renal homogenates of CTX-treated mice. As shown in Table 1, CTX caused oxidative stress in the kidneys by significantly increasing the MDA content and by decreasing antioxidant enzyme activities (*p* < 0.01 vs. control). In contrast, post-treatment with SCHPs-F1 for 15 consecutive days significantly increased the activities of antioxidant enzymes and reduced the MDA content in renal tissue. The high levels of MDA (20.88 ± 0.85 nmol/mg prot) and the low activities of antioxidant enzymes (CAT, 36.63 ± 1.23 U/mg prot; SOD, 10.60 ± 0.81 U/mg prot; GSH-Px, 10.07 ± 0.24 U/mg prot; T-AOC, 1.83 ± 0.11 U/mg prot) were ameliorated in mice treated with SCHPs-F1 400 mg/kg, showing a significant difference compared with the CTX-only group (*p* < 0.01). The levels of MDA and SOD almost returned to normal levels in mice treated with SCHPs-F1 400 mg/kg (*p* > 0.05 vs. control).

### 3.4. SCHPs-F1 Treatment Mitigates CTX-Induced Renal Inflammation

**Figure 4:**
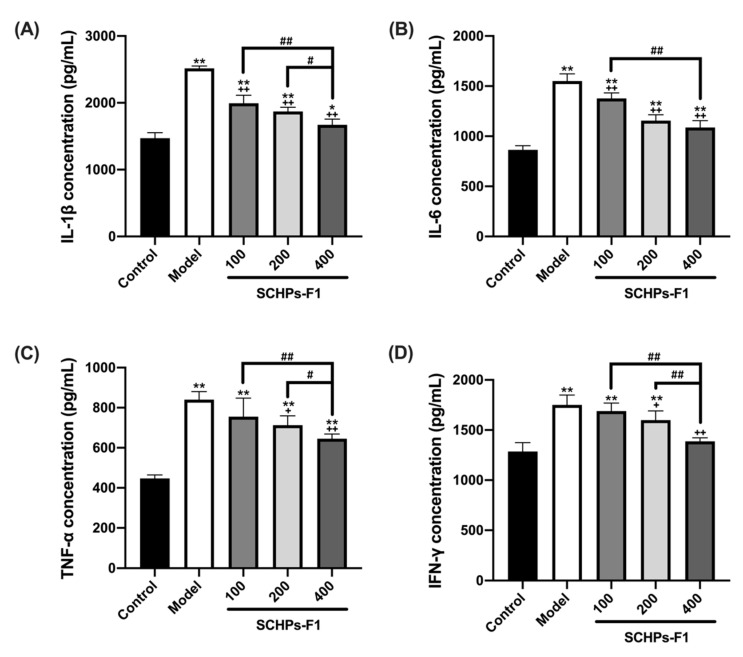
Effects of SCHPs-F1 on inflammatory cytokines levels of IL-1β (**A**), IL-6 (**B**), TNF-α (**C**), and IFN-γ (**D**) in CTX-exposed kidney tissues. * *p* < 0.05, ** *p* < 0.01, vs. control group; + *p* < 0.05, ++ *p* < 0.01, vs. model group; # *p* < 0.05, ## *p* < 0.01 indicate significant differences between different SCHPs-F1 dose groups.

The effect of SCHPs-F1 on nephritis was investigated by ELISA assays. The pro-inflammatory cytokine levels in mice following CTX induction showed a significant increase in IL-1β, IL-6, TNF-α, and IFN-γ in renal homogenates when compared to control mice (Figure 4). However, these negative changes caused by CTX were significantly attenuated by SCHPs-F1 treatment in a dose-dependent manner. As shown in Figure 4, the levels of IL-1β, IL-6, IFN-γ, and TNF-α (2517.14 ± 34.67, 1551.43 ± 71.56, 1750.65 ± 99.06 and 840.44 ± 40.28 pg/mL, respectively) were decreased at a SCHPs-F1 dose of 400 mg/kg (*p* < 0.01).

### 3.5. SCHPs-F1 Reverses Bax/Bcl-2 Imbalance in CTX-Exposed Kidney

**Figure 5:**
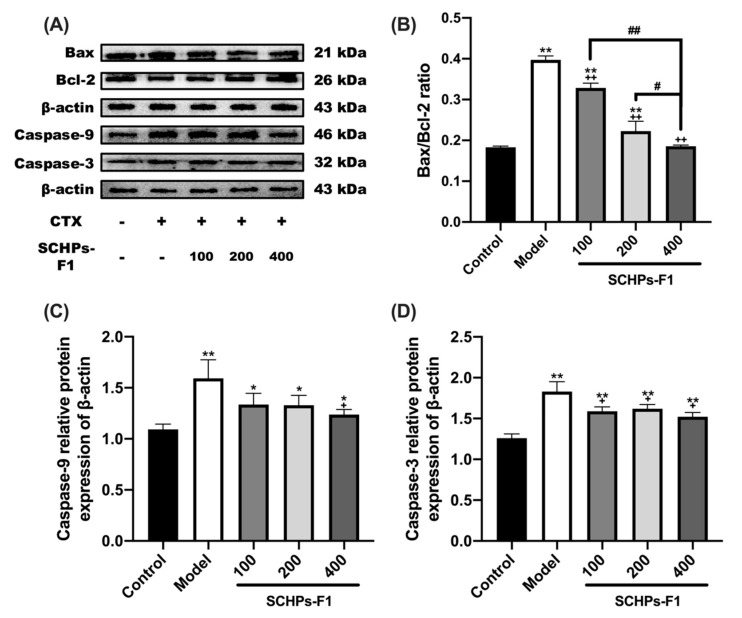
Effects of SCHPs-F1 treatment on CTX-induced changes in Bax/Bcl-2 (**A**,**B**) ratio, caspase-3 (**A**,**C**) and caspase-9 (**A**,**D**) expression in kidney tissue. * *p* < 0.05, ** *p* < 0.01, vs. control group; + *p* < 0.05, ++ *p* < 0.01, vs. model group; # *p* < 0.05, ## *p* < 0.01 indicate significant differences between different SCHPs-F1 dose groups.

CTX-induced inflammatory responses and oxidative stress are the main causes of nephrocyte apoptosis and renal function impairment [6,9]. To evaluate the effect of SCHPs-F1 on nephrocyte apoptosis in CTX-induced mice, we determined the expression of caspase-3 and caspase-9, and the ratio between the pro-apoptotic protein Bax and the anti-apoptotic protein Bcl-2 by Western blot analysis (Figure 5). Similar to inflammation and oxidative stress, the Bax/Bcl-2 ratio was significantly perturbed CTX-exposed renal tissue. Additionally, CTX treatment increased the expression of the apoptosis effector caspase-3 and apoptosis initiator caspase-9 in kidney tissue. On the other hand, post-treatment with SCHPs-F1 increased the expression of Bcl-2 and limited the overexpression of Bax, caspase-3, and caspase-9.

**Figure 6:**
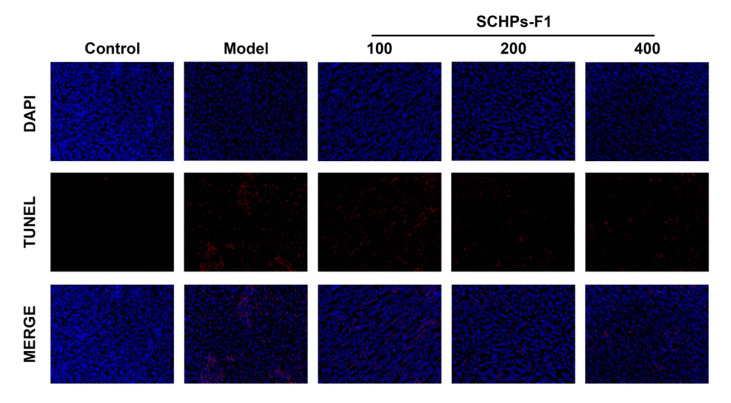
Effects of SCHPs-F1 on apoptosis in kidney tissues of CTX-treated mice assessed by TUNEL assay (×200 magnification). The top panels show representative images of 4,6-diamidino-2-phenylindole (DAPI)-stained cell nuclei (blue). The middle panels show representative images of TUNEL-positive cells labeled with red fluorescent dye Alexa Fluor 640. The bottom panels show the merged fluorescence images.

We further examined the effect of SCHPs-F1 on nephrocyte apoptosis in CTX-treated mice using the terminal deoxynucleotidyl transferase dUTP nick end labeling (TUNEL) assay. In accordance with the results of Western blot analysis, there were more TUNEL-positive cells in CTX-treated kidneys (Figure 6). This massive renal cell apoptosis was substantially alleviated by SCHPs-F1 treatment, indicating that SCHPs-F1 had an inhibitory effect on CTX-induced renal apoptosis.

### 3.6. SCHPs-F1 Treatment Ameliorates Pathomorphology in CTX-Exposed Kidney

**Figure 7:**
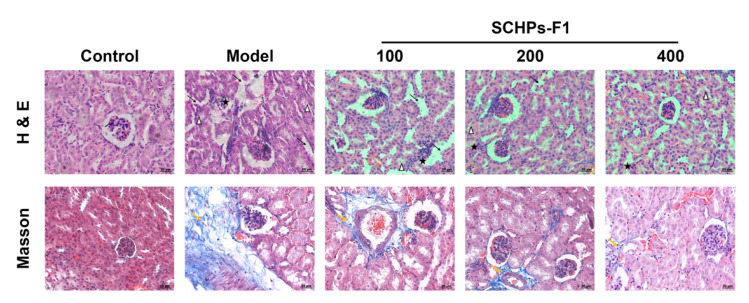
Effects of SCHPs-F1 treatment on the kidney pathomorphology in CTX-exposed mice (×400 magnification). H&E-stained images of kidney tissue paraffin sections showing inflammatory cell infiltrate (stars), mesangial matrix dilation (arrows), tubular necrosis and degeneration (arrowheads); Masson’s trichrome-stained images of kidney tissue paraffin sections showing blue-stained collagen fibrous connective tissue (yellow arrows).

To evaluate the effect of SCHPs-F1 on renal histopathology in CTX-treated mice, H&E staining was performed (Figure 7). In the control group, renal sections showed intact architectures of the glomerulus, proximal convoluted tubules, and distal convoluted tubules, and no inflammatory infiltrates were detected. In CTX-treated mice, the extensive and severe renal injury was observed, including mesangial matrix dilation, tubular necrosis and degeneration, and infiltration by inflammatory cells. However, these histopathological lesions were effectively ameliorated following treatment with SCHPs-F1. Further, Masson trichrome staining was used to observe the effect of CTX and SCHPs-F1 treatment on the severity of renal fibrosis in mice. Compared with control mice, CTX-treated mice showed extensive blue collagen fibrous connective tissue, with a severe degree of renal fibrosis. When mice were treated with SCHPs-F1, the area of blue-stained collagen fibers decreased, indicating that the severity of renal fibrosis was reduced.

### 3.7. SCHPs-F1 Elevate Nrf2 Related Protein Expression in CTX-Induced Kidney

**Figure 8:**
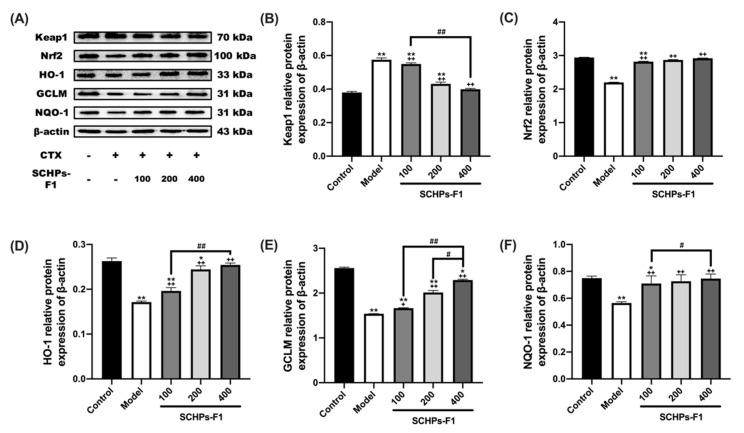
SCHPs-F1 treatment down-regulated Keap1 (**A**,**B**) and up-regulated Nrf2 (**A**,**C**), HO-1 (**A**,**D**), GCLM (**A**,**E**), and NQO-1 (**A**,**F**) protein levels in CTX-induced renal. * *p* < 0.05, ** *p* < 0.01, vs. control group; + *p* < 0.05, ++ *p* < 0.01, vs. model group; # *p* < 0.05, ## *p* < 0.01 indicate significant differences between different SCHPs-F1 dose groups.

The effect of SCHPs-F1 on the expression of Nrf2 pathway-related proteins in the kidneys of CTX-induced mice was investigated by Western blot analysis (Figure 8). The data demonstrated that the expression of repressor protein Keap1 was increased with CTX treatment when compared with the Control group (*p* < 0.01). SCHPs-F1 treatment significantly decreased Keap1 protein expression and up-regulated Nrf2 protein levels in CTX-induced mice. In addition, the downstream antioxidant proteins HO-1, GCLM, and NQO-1, which are associated with Nrf2 protein expression, were also notably up-regulated compared with mice that were treated with SCHPs-F1 only. Together, these results revealed that SCHPs-F1 were able to mitigate renal oxidative stress following CTX-induction by increased Nrf2 expression and by up-regulating the activities of HO-1, GCLM, and NQO-1.

## 4. Discussion

The kidneys are important metabolic organs in mammals; they excrete metabolic waste and chemical metabolites by filtering urine and maintain homeostasis and normal physiological activities [21]. Several studies have shown that the nephrotoxicity of CTX metabolites is a serious limitation in cancer chemotherapy [9,22]. In this study, SCHPs-F1 presented antioxidant activity *in vitro* including radical scavenging and reducing power. Interestingly, it has been reported that SCHPs-F1 ameliorated hepatotoxicity by reducing the oxidative stress status of liver in CTX-induced mice [14]. In the present study, we investigated the effect of SCHPs-F1 on CTX-induced nephrotoxicity in a mouse model. The results indicate that treatment with SCHPs-F1 after exposure to CTX ameliorated inflammation responses and reduced oxidative stress and apoptotic markers in renal tissue. This attenuating effect of SCHPs-F1 may be related to the activation of the Nrf2 antioxidant signaling pathway.

In the present study, the observed increase in the renal index, BUN, and CRE levels and the decrease in CYP450 content suggested CTX-induced renal function impairment and renal damage, as demonstrated in previous studies [23,24,25]. BUN and CRE are excreted through glomerular filtration. Decreased glomerular filtration leads to increased BUN and CRE levels, which are measures of the glomerular filtration rate, and thus of renal function [26,27]. Moreover, renal function can also be interpreted by assessing CYP450 content [24,28]. Acrolein, a toxic metabolite of CTX, which combines with free sulfhydryl groups to inactivate the CYP450 metabolizing enzyme, results in a decrease of total CYP450 content [1,29]. Histopathological findings confirmed these results, including severe renal structural damage, such as necrosis and exfoliation of endothelial cells and basement membrane epithelial cells, infiltration by inflammatory cells, and interstitial hemorrhage. Moreover, Masson’s trichrome staining suggested that CTX caused severe fibrosis in renal tissue. Notably, CTX-induced renal dysfunction and pathological changes in renal tissue were attenuated after post-treatment with SCHPs-F1, as supported by the observed trends in BUN, CRE, and CYP450 levels that recovered to normal levels.

The present data thus indicate that SCHPs-F1 treatment significantly relieved CTX-induced renal oxidative stress, inflammatory responses, and apoptosis in mice, likely by enhancing the antioxidant protective system comprising GSH-Px, SOD, CAT, and T-AOC, and by suppressing the lipid peroxidation product MDA, the pro-inflammatory cytokines IL-1β, IL-6, TNF-α and IFN-γ, and the pro-apoptotic Bax/Bcl-2 ratio. It is known that acrolein has a severe adverse effect on the kidneys, which results in the robust activation of oxidative stress responses and further aggravation of renal injury [1,30,31]. GSH-Px, SOD, CAT, and T-AOC are critical indicators for evaluating the capacity of the cellular antioxidant defense system [32,33]. An imbalance in oxidative stress defense leads to insufficient antioxidant responses and lipid peroxidation to generate MDA [31,34]. As mentioned, SCHPs-F1 increased the antioxidant defense parameters GSH-Px, SOD, CAT, and T-AOC and decreased levels of the lipid peroxidation marker MDA, thereby potentially alleviating oxidative stress of CTX-exposed kidneys. A similar mechanism to enhance the endogenous antioxidant defense to alleviate CTX-induced renal injury was reported for aminoguanidine [35], *Olea europaea* leaf extract [6], and plasma protein from *Tachypleus tridentatu* [21].

Oxidative stress signaling cascades are closely related to inflammatory responses and apoptosis in the kidneys [36,37,38]. The increase in pro-inflammatory cytokines may relate to the damage of renal cell structure caused by oxidative stress and lipid peroxidation. Consistently, previous studies revealed that CTX-induced nephrotoxicity resulted in the enhancement of pro-inflammatory cytokines [7,26,39]. However, numerous studies supported that inflammatory factor inhibition plays an indispensable role in the prevention of CTX-mediated renal injury [6,37]. In the present study, treatment with SCHPs-F1 reversed the CTX-induced increases in the kidney’s levels of IL-1β, IL-6, TNF-α, and IFN-γ. The mitochondrial-dependent apoptotic pathway induces apoptosis by perturbing the balance between apoptotic protein Bax and anti-apoptotic protein Bcl-2 and inducing the expression of caspase proteins [36,40]. Consistent with previous reports on renal cell apoptosis [21,41], our results showed that the increase in the Bax/Bcl-2 ratio, and the activation of caspase-3 and caspase-9 in the kidney were suppressed by treatment with SCHPs-F1 after exposure to CTX. The results of the TUNEL assay showed that SCHPs-F1 treatment had a certain protective effect on renal apoptosis in CTX-induced mice.

The Keap1-Nrf2-antioxidant response element (ARE) signaling pathway is an oxidative stress-sensitive defensive response system [42,43]. Upon exposure to adverse environmental pressure, including many exogenous chemicals, the transcription factor Nrf2 escapes inhibition by the specific repressor Keap1 in the cytoplasm and associates with AREs in the nucleus where it activates the expression of multiple antioxidant enzymes and cytoprotective proteins that contribute to the defense against oxidative stress and alleviate cell damage [6,44]. Therefore, the expression of Nrf2 and its downstream antioxidant-related gene products HO-1, GCLM, and NQO-1 were investigated by means of Western blot analysis. Our results suggest that SCHPs-F1 treatment after CTX exposure had a de-repressive effect on the Keap1-Nrf2-ARE pathway. SCHPs-F1 restored the CTX-suppressed expression of HO-1, GCLM, and NQO-1, indicating that activation of the Keap1-Nrf2 signaling pathway may be involved in SCHPs-F1-mediated amelioration of CTX-induced oxidative stress, inflammation, apoptosis, and renal injury.

## 5. Conclusions

In the present study, we demonstrated that post-treatment with SCHPs-F1 efficiently attenuated CTX-induced renal injury and this correlated with decreased oxidative stress, inflammatory responses, and cell apoptosis, which may be mediated by activation of Nrf2 antioxidant signaling. Our results indicate that treatment with SCHPs-F1 alleviated renal injury and renal dysfunction caused by CTX, as manifested by a decrease in the renal index, an amelioration of pathological morphology, and restoration of BUN, CRE, and CYP450 levels. In addition, SCHPs-F1 induced a decrease in oxidative stress, inflammatory responses, and apoptosis markers. Taken together, these findings suggest that of SCHPs-F1 (and active substances contained therein) hold promise for the restoration of renal dysfunction induced by CTX.

## Figures and Tables

**Table 1:** The effects of SCHPs-F1 on MDA, CAT, SOD, GSH-Px, and T-AOC levels in renal homogenates o CTX-induced mice.

	MDA (nmol/mg Prot)	CAT (U/mg Prot)	SOD (U/mg Prot)	GSH-Px (U/mg Prot)	T-AOC (U/mg Prot)
Control	18.67 ± 1.31	39.28 ± 1.18	11.31 ± 0.25	11.15 ± 0.33	2.08 ± 0.09
Model	34.01 ± 2.23 **	30.65 ± 1.29 **	7.62 ± 0.63 **	8.44 ± 0.43 **	1.16 ± 0.05 **
SCHPs-F1 100	28.92 ± 1.36 **,++	32.52 ± 2.20 **	8.37 ± 0.19 **	8.66 ± 0.42 **	1.35 ± 0.09 **,+
SCHPs-F1 200	26.64 ± 1.29 **,++	35.25 ± 2.45 *,++	8.83 ± 0.64 **,+	8.79 ± 0.11 **	1.54 ± 0.04 **,++
SCHPs-F1 400	20.88 ± 0.85 ++	36.63 ± 1.23 **,++	10.60 ± 0.81 ++	10.07 ± 0.24 **,++	1.83 ± 0.11 **,++

* *p* < 0.05, ** *p* < 0.01, vs. control group; + *p* < 0.05, ++ *p* < 0.01, vs. model group.
